# Review of *Anopheles* Mosquito Species, Abundance, and Distribution in Ethiopia

**DOI:** 10.1155/2021/6726622

**Published:** 2021-09-23

**Authors:** Fasil Adugna, Melaku Wale, Endalkachew Nibret

**Affiliations:** ^1^Department of Biology, Bahir Dar University, P.O. Box 79, Bahir Dar, Ethiopia; ^2^Biotechnology Research Institute, Bahir Dar University, P.O. Box 79, Bahir Dar, Ethiopia

## Abstract

**Background:**

Malaria is a major mosquito-borne disease in Ethiopia, and it is one of the leading causes of morbidity and mortality. *Plasmodium falciparum* and *P. vivax* are the two malaria-causing parasitic species commonly known to cause human malaria in Ethiopia. To better manage and control vectors transmitting malaria parasites, the abundance, distribution, and updated annotated list of *Anopheles* species present in Ethiopia are very important.

**Methods:**

In order to compile a list of the species recorded in Ethiopia, 33 original research articles were collected. This work gives an updated list of *Anopheles* mosquito species in Ethiopia and their abundance, distribution, and composition.

**Results:**

According to this review, 110305 *Anopheles* mosquitoes were collected and 35 *Anopheles* species were recorded in different parts of Ethiopia. *A. arabiensis* was the most abundant when compared to other species, whereas *A. maculipalpis* and *A. wilsonii* were the least abundant species. The most abundant *Anopheles* species was recorded in central and the least abundant, from eastern Ethiopia. The second, third, and fourth abundant species were also collected from southern, northern, and western parts of Ethiopia.

## 1. Background

Ethiopia is located in the eastern part of the African continent, known as the Horn of Africa. It is the oldest independent country in Africa. The country is bounded by Eritrea in the north, Djibouti in the northeast, Somalia in the east, Kenya in the south, South Sudan in the west, and Sudan in the northwest. Ethiopia lies between the equator and northern tropic between the 3°N and 15°N latitude or 33°E and 48°E longitude. Ethiopia is the most populous landlocked country in the world and the second-most populous nation on the African continent. The country has a total area of 1,100,000 square kilometers (420,000 square mile). Its capital and the largest city is Addis Ababa, located almost at the center of the country.

Ethiopia is endowed with different types of plants and animals including mosquito species. The interaction of mountain landscapes with other variables, such as winds, seasonal rains, and temperatures, creates diverse environmental conditions for malaria transmission. These conditions create local puddles, flooding, and warm ambient temperatures, which are suitable for *Anopheles* mosquito breeding, and may accelerate focal malaria transmission.

Mosquitoes belong to the phylum Arthropoda, class Insecta, and order Diptera [1]. They are divided into three subfamilies: Toxorhynchitinae, Anophelinae, and Culicinae [2]. There are over 3,530 species of mosquitoes in the world which are grouped under 43 genera; all these genera are included in the Culicidae family [3]. From these genera, *Anopheles*, *Aedes*, and *Culex* are medically important due to their ability to transmit mosquito-borne human diseases [4]. Some mosquito species bite humans routinely and act as vectors of a number of infectious diseases affecting millions of people annually in Sub-Saharan Africa [5, 6].

Mosquitoes are regarded as public enemies because of their biting annoyance, noise nuisance, and cause of sleeplessness. In addition to this, they can transmit disease-causing agents and lead to human diseases such as malaria, yellow fever, chikungunya, lymphatic filariasis, dengue, hemorrhagic fever and encephalitis [7, 8], diseases caused by Zika virus and filariasis, and eventually cause high morbidity and mortality in human [9]. Moreover, mosquitoes can transmit animal diseases such as the fowl-pox of poultry, myxomatosis of rabbits, rift-valley fever of sheep, encephalitis of horses and birds, and heartworm diseases of dogs [10].

Malaria is a major mosquito-borne disease in Ethiopia, and it is one of the leading causes of morbidity and mortality. *P. falciparum* and *P. vivax* are the two parasitic species commonly known to cause malaria in Ethiopia accounting for 60% and 40% proportion, respectively [11]. The disease is transmitted by the bites of infective female *Anopheles* mosquitoes [12–14].

In Ethiopia, more than forty species of *Anopheles* mosquitoes have been documented so far [15–18]. Of all *Anopheles* mosquito species, *A. arabiensis*, *A. funestus*, *A. pharoensis*, and *A. nili* are the medically important malaria vectors in Ethiopia [19–21]. Among these, *A. arabiensis* is a principal malaria vector largely distributed in Ethiopia [22]. The other common vectors of malaria dominating in malaria-endemic areas are *A. funestus* and *A. pharoensis* [23–25]. *Anopheles nili* is the least common and more localized species, and it is not adequately studied. It is found in the southwestern, western, and northwestern parts of Ethiopia [20, 23]. Recently, *A. stephensi* was identified in the eastern Ethiopia [26].

Ethiopia is a country that has implemented revised strategies to control malaria. The major vector control methods currently implemented in malaria vector programs include methods targeting larvae and adult mosquitoes. Among these, indoor residual spraying (IRS), long-lasting insecticidal nets (LLINs), and larval source management (LSM) are the most important mosquito vector control approaches [27]. IRS and LLINs are used to reduce the density, feeding frequency, and longevity of malaria vectors by killing the vectors with insecticides or blocking their contact with humans [28, 29] and primarily target malaria vectors that feed and rest inside the house at night [30]. In addition to these, community education, introductions of rapid diagnostic tests, and adaptation of artemisinin-based combination therapies (ACTs) are also practiced [31]. On the other hand, the developments of insecticide and drug resistance in different insecticide groups and drugs have their own impact on the control of the main malaria vectors. In Ethiopia, different researchers [27, 32, 33] reported the development of resistance in different insecticide groups on *A. arabiensis*.

In Ethiopia, a large number of *Anopheles* mosquitoes were tested for *Plasmodium* circumsporozoite protein (CSP) using ELISA. However, a few studies conducted in different parts of Ethiopia (Ghibe River Basin [34], Bahir Dar Zuria District [35], Sekoru District [36], Meskan and Mareko district [37], Gilgel-Gibe dam area [38], Arba Minch area [39], and Gamo Gofa zone [40]) showed the presence of sporozoite infection on principal vectors (*A. arabiensis* and *A. pharoensis*).

One old study showed the existence of 34 *Anopheles* mosquito species and two subspecies in different provinces of Ethiopia [41]. Two recent studies also showed the presence of more than 40 *Anopheles* species in the country [17, 18], but these studies did not clearly show the abundance and exact locations of each species. An updated list of *Anopheles* species documenting their abundance and distribution is very important to get information easily for better management and control of mosquitoes. Therefore, the aim of this review was to produce a new and recent list of all *Anopheles* mosquito species with specific information on their abundance and geographic distribution that have been reported up to February 2020 in Ethiopia.

## 2. Methods

The available published and unpublished studies on the *Anopheles* mosquito were collected and reviewed. Articles published starting from 1990 to February 2020 were extracted. Related articles were searched using search engines from the following electronic bibliographic databases: PubMed, Google Scholar, and Science Direct literature in order to identify studies conducted on the diversity and composition of *Anopheles* mosquitoes. In addition, manual Google searching and screening of reference lists were done to access additional articles.

Articles were searched without time restriction. Articles were searched using the key terms “*Anopheles* mosquito diversity,” “composition,” and “abundance and density in Ethiopia.” The searched articles should describe the original collections of *Anopheles* mosquitoes, which were identified to the species level. Studies only citing another article's collections (second-hand accounts) were not included. All articles were entered into a spreadsheet to facilitate the calculation of the number of species and the number of records for each species.

## 3. Results

### 3.1. Abundance and Composition of *Anopheles* Species

In this paper, 33 original research articles that were conducted in different parts of Ethiopia were reviewed. Most of original research articles (*n* = 26) were reviewed in Oromia Regional State, whereas the least were Gambella and Somali Regional States (*n* = 1). The original studies were focused on larvae, adults, and a combination of both. The larvae were collected using a standard dipper, whereas the adults were collected using different collecting methods such as human-biting catches, light traps, pit traps, pyrethrum spray catch, and aspirator. For this review, different studies conducted indoor, outdoor, or in both locations were recorded (Table S1).

**Figure 1:**
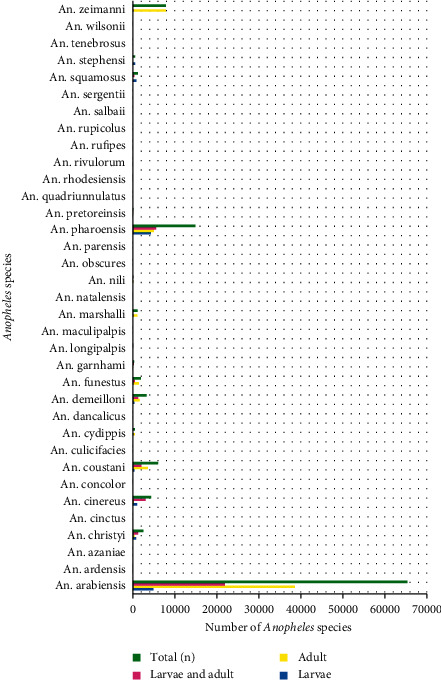
List of *Anopheles* species and their abundance in Ethiopia.

**Figure 2:**
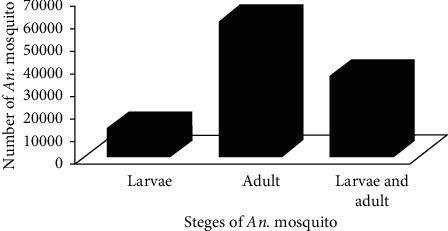
Total number of *Anopheles* species in Ethiopia.

According to this review paper, a total of 110305 *Anopheles* mosquitoes were collected and 35 *Anopheles* species were recorded in different parts of Ethiopia. These species are listed alphabetically in Figure 1. Of the total *Anopheles* mosquitoes collected, the adult was more abundant (*n* = 60759) than larvae (*n* = 13150) (Figure 2). From the total *Anopheles* species collected, *A. arabiensis* was the most abundant (*n* = 65389) when compared to other species, whereas *A. maculipalpis* and *A. wilsonii* were the least abundant (*n* = 1) for each species. The second rank was occupied by *A. pharoensis (n* *=* 14874). In addition to this, *A. arabiensis* was more abundant (*n* = 38579) in its adult stage than in other stages (Figure 1).

### 3.2. Geographical Distribution of *Anopheles* Mosquito in Ethiopia

**Figure 3:**
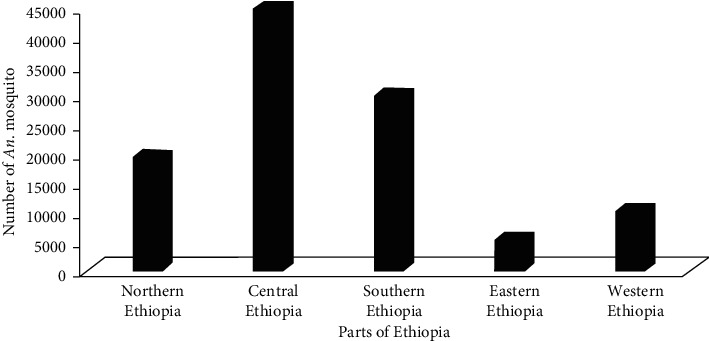
Distributions of *Anopheles* species in different parts of Ethiopia.

**Figure 4:**
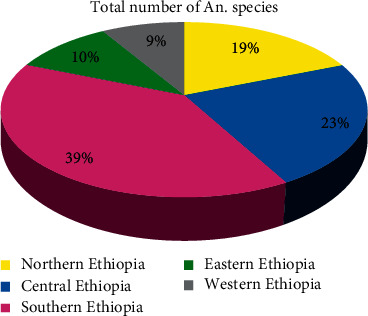
Percentage of *Anopheles* species in different parts of Ethiopia.

The collected *Anopheles* mosquitoes were distributed in northern, central, eastern, and western parts of Ethiopia. The most abundant *Anopheles* mosquitoes (*n* = 44935**)** were recorded in central Ethiopia, whereas the least (*n* = 5435**)** were recorded in the eastern parts of Ethiopia. The second and third abundant *Anopheles* mosquitoes were collected in central (*n* = 44935) and southern (*n* = 30076) parts of Ethiopia (Figure 3). Furthermore, the largest number and percentage of *Anopheles* species (*n* = 27 (39%)) were recorded in southern Ethiopia, while the least number and percentage (*n* = 6 (9%)) were recorded in the western parts of Ethiopia. The second (*n* = 16 (23%)), third (*n* = 13 (19%)), and fourth (*n* = 7 (10%)) and the largest number of *Anopheles* species were also found in central, northern, and eastern parts of Ethiopia (Figure 4). Moreover, *A. arabiensis*, *A. coustani*, and *A. pharoensis* were recorded in five directional locations of Ethiopia.

### 3.3. Frequency of Occurrence of *Anopheles* Species

**Figure 5:**
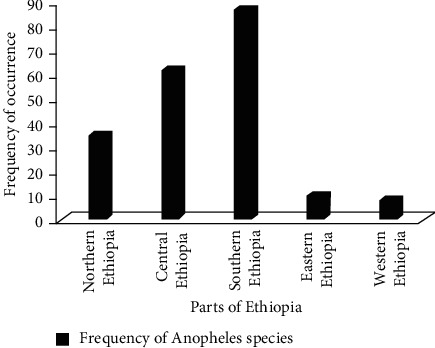
Total frequency and abundance of *Anopheles* species in different parts of Ethiopia.

**Figure 6:**
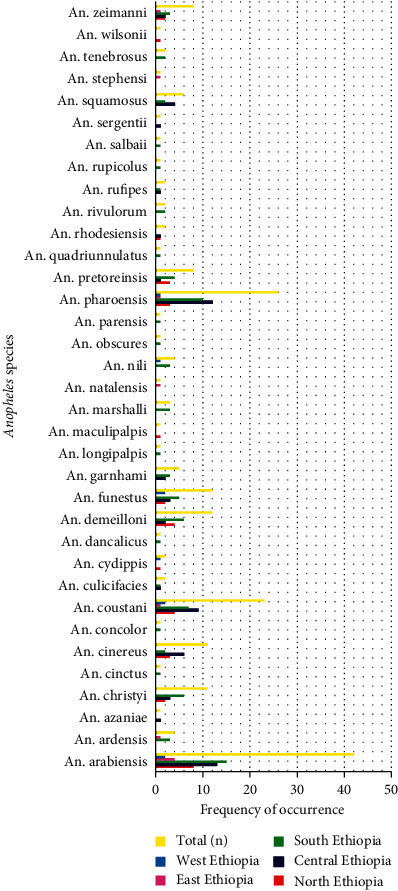
Frequency of occurrence of *Anopheles* species in different parts of Ethiopia.

The most abundant and frequently occurring *Anopheles* mosquitoes (*n* = 87**)** were recorded in the southern parts of Ethiopia, while the least (*n* = 8**)** were recorded in the eastern parts of Ethiopia. The second (*n* = 62), third (*n* = 35), and fourth (*n* = 10) ranks were occupied by central, northern, and eastern parts of Ethiopia, respectively (Figure 5). In addition to this, *A. arabiensis* was the most frequently (*n* = 42) occurring *Anopheles* species in Ethiopia. The highest frequently occurring *A. arabiensis* (*n* = 15) was found in southern Ethiopia, whereas the least (*n* = 2) frequently occurring was found in western parts of Ethiopia. The second (*n* = 26) and third (*n* = 23) frequently occurring species were also *A. pharoensis* and *A. coustani,* respectively. On the other hand, *A. azaniae, A. cinctus*, *A. concolor, A. dancalicus, A. culicifacies, A. longipalpis, A. maculipalpis, A. natalensis, A. obscures, A. parensis*, *A. wilsonii, A. quadriunnulatus, A. rufipes, A. rupicolus, A. salbaii*, *A. sergentii*, and *An. stephensi* were the least (*n* = 1) frequently occurring species. They were found in only one location in Ethiopia (Figure 6).

## 4. Discussion

Recent information on *Anopheles* mosquito abundance and composition in Ethiopia is important. There have been some changes in taxonomic classification and nomenclature in recent years. On the other hand, the names of species have been changed, or subspecies might take another taxonomic rank. In any case, new collections have shown new species and adding new species to the previous list of *Anopheles* mosquitoes in Ethiopia.

In the present review paper, 35 *Anopheles* species were recorded in different parts of Ethiopia by reviewing 33 original research articles. On the other hand, one entomological report back in 1967 showed the presence of 34 species and two subspecies of *Anopheles* in different provinces of Ethiopia [41]. When the oldest and the present lists of *Anopheles* species were compared, only 21 species were found in both lists. However, 14 *Anopheles* species, namely, *A. arabiensis, A. azaniae*, *A. concolor*, *A. culicifacies*, *A. zeimanni*, *A. sergentii*, *A. stephensi*, *A. tenebrosus*, *A. wilsonii*, *A. quadriunnulatus, A. salbaii*, *A. parensis*, *A. cydippis*, and *A. cinctus,* were recorded in our current list, but not in the old one.

The appearance of these new species may be due to changes in the taxonomic classification and nomenclature in recent years using molecular techniques in Ethiopia. Moreover, *A. gambiae* complex speciation occurs in different ecological niches as result of climate change [42, 43], gene exchanges between species (gene flow) [44], paracentric chromosomal inversions [43], and evolution of reproductive isolation by divergent natural selection [45] and their transportation with items such as cans and tyres from neighboring countries may add new species to the list. For example, *A. gambiae* in the old list is now changed into *A. arabiensis* in the new list. A recent study conducted in the Somali region, Kebri Dehar district, confirmed the presence of *A. stephensi (*which might be introduced into Ethiopia from neighboring countries) by molecular approach for the first time in Ethiopia [46]. On the other hand, 13 *Anopheles* species recorded in the old list were not found in the new list. This is because of the lack of entomological studies at each specific site of Ethiopia, and the researchers generally depend on the disease-causing *Anopheles* mosquitoes. Of all *Anopheles* mosquito species, *A. arabiensis, A. funestus*, *A. pharoensis*, and *A. nili* were the medically important malaria vectors in Ethiopia with decreasing order of importance [19–21].

More adults (*n* = 60759) were found than other stages, and larvae were the least abundant (*n* = 13150). The reason behind this is the adult stage was collected both indoor and outdoor locations by using different collection methods such as human-biting catches, light traps, pit trap, pyrethrum spray catch, and aspirator, while the larval stage was collected only using a standard dipper. In addition to this, most of the studies (*n* = 15) focused on the adult stage of *Anopheles* because adults were relatively simple to process, manage, and identify (Table S1).

From the total *Anopheles*, *A. arabiensis* and *A. pharoensis* were the first and the second most abundant species when compared to other species. This is similar to other studies conducted in Ethiopia. *A. pharoensis* is the second dominating vector of malaria next to *A. arabiensis* in malaria-endemic areas of Ethiopia [23–25]. The study conducted in central Ethiopia rift valley showed that the larvae of *A*. *arabiensis* and *A. pharoensis* were the most abundantly sampled species [47]. In addition to this, *A. arabiensis* was the dominant species in highland and lowland areas of Dirashe district, south Ethiopia [48, 49].

The most abundant *Anopheles* mosquitoes (*n* = 44935) were recorded in central Ethiopia, whereas the least (*n* = 5435**)** were recorded in the eastern parts of Ethiopia. The reason for this is central Ethiopia is close to Addis Ababa city; hence, this area is rich in electric power dam, drinking water reserve, presence of natural rift valley, and irrigation canal. For example, *Anopheles* mosquitoes were studied in different sites of central Ethiopia, such as in the shoreline of Koka reservoir [50], irrigation and major drainage areas in the middle course of the Rift Valley [47], small-scale irrigation scheme and water canal around Zeway [51, 52], and irrigation and major drainage areas between Adami Tulu and Meki towns [53]. Therefore, this area is suitable for a breeding place of mosquito and responsible for this abundance. In addition to this, central Ethiopia is close to Addis Ababa University and other different research centers. Consequently, many researchers are attracted in these areas in order to partially fulfill the requirements for the Degree of Masters or Ph.D.

On the other hand, the southern part of Ethiopia receives rain throughout the year, and it has the highest temperature, which is suitable for breeding and development of mosquitoes. In addition to this, southern Ethiopia is rich in irrigation development sites, banana and “*enset”* plantation, natural lakes, and river basins. For example, *Anopheles* mosquitoes were studied in different parts of southern Ethiopia, such as Ghibe River Basin [54], the Arjo-Dedessa irrigation development site [55], Gamo Gofa Great Rift Valley Lake [56], and different agro-ecology such as irrigation and agricultural areas [36]. Due to such reasons, a southern part of Ethiopia takes the second rank in terms of hosting most *Anopheles* mosquitoes in the country. *A. arabiensis* were recorded in all directions of Ethiopia, but the highest abundance was recorded in the southern part of Ethiopia. This is similar to a study conducted by [22].

## 5. Conclusions

However, the mosquito taxonomy studies are still not sufficient in Ethiopia since there are no recent and proper published morphological identification keys available for local *Anopheles* mosquito species. In addition, most entomological surveys conducted in Ethiopia did not consider all *Anopheles* mosquito species, other than medically important ones. Therefore, the abundance, composition, and distribution of *Anopheles* mosquito fauna have received very limited attention. This may ultimately hinder noticing the appearance of new species or nonrecorded species from the country. Hence, disease surveillance programmers should characterize the species they encounter in collections and the development of morphological identification keys to the locally available mosquitoes.

The present systematic review provides the most updated list of *Anopheles* mosquito species and their abundance, composition, and distribution of *Anopheles* mosquito fauna in Ethiopia. Further work is required to refine this list and understand the distributions and abundance of those species in regional, zonal, and district level within the country. In addition to this, it should give appropriate justifications for the presence of unequal distribution and abundance of *Anopheles* mosquitoes in different parts of Ethiopia. This may serve as a basis for the development of identification keys for other mosquito genera and will act as a catalyst to study on bionomics of these species.

## Data Availability

The raw data supporting the conclusion of this study are accessible from the corresponding author.

## Supplementary Materials

Supplementary Materials Table S1: List of *Anopheles* mosquitoes found in different parts of Ethiopia.
