# Supplementary material for: Review of Anopheles Mosquito Species, Abundance, and Distribution in Ethiopia
Source: J Trop Med. 2021 Sep 23;2021:6726622. doi: 10.1155/2021/6726622 (PMC8486561; doi:10.1155/2021/6726622)
Supplement: Supplementary Materials — Table S1: List of Anopheles mosquitoes found in different parts of Ethiopia. [file 6726622.f1.doc]

**Table S1. List of *Anopheles* mosquito found in different parts of Ethiopia**

| **No.** | **Species** | **Stage** | **Number** | **Collection methods** | **Study area** | **Region** | **Collected place** | **Author** |
| --- | --- | --- | --- | --- | --- | --- | --- | --- |
| 1 | *An. stephensi* | Larvae | 535 | Dipping | Kebri Dehar, east Ethiopia | Somali | outdoor |  |
| 2 | *An. arabiensis* | Larvae | 604 | Dipping | Koka reservoir in central Ethiopia | Oromia | outdoor |  |
|  | *An. pharoensis* |  | 1097 |  |  |  |  |  |
|  | *An. Coustani* |  | 58 |  |  |  |  |  |
|  | *An. squamosus* |  | 38 |  |  |  |  |  |
| 3 | *An. quadriunnulatus* | Adult | 29 | Hand aspirator | Jimma (south-west Ethiopia) | Oromia | Indoor/outdoor |  |
|  | *An. arabiensis* |  | 29 |  |  |  |  |  |
| 4 | *An. arabiensis* | Adult | 3724 | Light traps, PSC and pit trap | Chano (north of Arba Minch) | SNNP | Indoor/outdoor |  |
|  | *An. marshalli* |  | 821 |  |  |  |  |  |
|  | *An. garnhami* |  | 61 |  |  |  |  |  |
|  | *An. funestus* |  | 26 |  |  |  |  |  |
|  | *An. pharoensis* |  | 21 |  |  |  |  |  |
| 5 | *An. arabiensis* | Larvae and adult | *921* | Dipping and PSC | Jabi Tehnan District, West Gojjam Zone | Amhara | Indoor |  |
|  | *An. cinereus* |  | 76 |  |  |  |  |  |
|  | *An. chrysti* |  | 40 |  |  |  |  |  |
|  | *An. demeilloni* |  | 30 |  |  |  |  |  |
|  | *An. rhodesiensis* |  | 18 |  |  |  |  |  |
| 6 | *An. arabiensis* | Larvae/ Pupa | 3329 | Dipping | Ghibe River Basin (Guraghe Zone) | SNNP |  |  |
|  | *An. pharoensis* |  | 39 |  |  |  |  |  |
|  | *An. christyi* |  | 101 |  |  |  |  |  |
|  | *An. rivulorum* |  | 6 |  |  |  |  |  |
|  | *An. demeilloni* |  | 1 |  |  |  |  |  |
|  | *An. pretoriensis* |  | 2 |  |  |  |  |  |
|  | *An. Coustani* |  | 3 |  |  |  |  |  |
|  | *An. nili* |  | 1 |  |  |  |  |  |
|  | *An. Concolor* |  | 2 |  |  |  |  |  |
|  | *An. ardensis* |  | 1 |  |  |  |  |  |
| 7 | *An. Arabiensis,* | Adult | 755 | Light trap | Highland of Dirashe  district | SNNP | Indoor |  |
|  | *An. demeilloni* |  | 347 |  |  |  |  |  |
|  | *An. cinereus* |  | 102 |  |  |  |  |  |
|  | *An. funestus* |  | 22 |  |  |  |  |  |
|  | *An. pharoensis* |  | 22 |  |  |  |  |  |
|  | *An. christyi* |  | 6 |  |  |  |  |  |
|  | *An. pretoriensis* |  | 8 |  |  |  |  |  |
|  | *An. ardensis* |  | 3 |  |  |  |  |  |
|  | *An. tenebrosus* |  | 3 |  |  |  |  |  |
| 8 | *An. arabiensis,* | Larvae | 686 | Dipping | Meki and Adami Tulu towns | Oromia | outdoor |  |
|  | *An. pharoensis* |  | 1015 |  |  |  |  |  |
|  | *An. squamosus* |  | 365 |  |  |  |  |  |
|  | *An. coustani* |  | 61 |  |  |  |  |  |
|  | *An. cinereus* |  | 7 |  |  |  |  |  |
| 9 | *An. cinereus,* | Larvae | 1008 | Dipping | Butajira area (south-central Ethiopia) | SNNP | outdoor |  |
|  | *An. arabiensis* |  | 973 |  |  |  |  |  |
|  | *An. chrysti* |  | 712 |  |  |  |  |  |
|  | *An. demeilloni* |  | 378 |  |  |  |  |  |
|  | *An. pretoriensis* |  | 17 |  |  |  |  |  |
|  | *An. azaniae* |  | 4 |  |  |  |  |  |
|  | *An. rufipes* |  | 3 |  |  |  |  |  |
|  | *An. sergentii* |  | 2 |  |  |  |  |  |
|  | *An. garnhami* |  | 2 |  |  |  |  |  |
|  | *An. pharoensis* |  | 1 |  |  |  |  |  |
| 10 | *An. arabiensis* | Adult | 2431 | Light traps, PSC and pit trap | Butajira area (south-central Ethiopia) | SNNP | Indoor/out door |  |
|  | *An. pharoensis* |  | 359 |  |  |  |  |  |
|  | *An. christyi* |  | 406 |  |  |  |  |  |
|  | *An. cinereus* |  | 163 |  |  |  |  |  |
|  | *An. demeilloni* |  | 1199 |  |  |  |  |  |
|  | *An. coustani* |  | 16 |  |  |  |  |  |
|  | *An. culicifacies* |  | 12 |  |  |  |  |  |
|  | *An. garnhami* |  | 3 |  |  |  |  |  |
|  | *An. rhodesiensis* |  | 1 |  |  |  |  |  |
| 11 | *An. arabiensis* | Adult | *6215* | PSC, light traps and pit trap | Bahir Dar Zuria District | Amhara | Indoor/outdoor |  |
|  | *An. pharoensis* |  | 1948 |  |  |  |  |  |
|  | *An. coustani* |  | 696 |  |  |  |  |  |
| 12 | *An. arabiensis* | Larvae and adult | 59 | Dipping, light trap, PSC and pit trap | Derashe District | SNNP | Indoor/outdoor |  |
|  | *An. christyi,* |  | 35 |  |  |  |  |  |
|  | *An. demeilloni* |  | 19 |  |  |  |  |  |
|  | *An. funestus* |  | 18 |  |  |  |  |  |
|  | *An. pharoensis* |  | 4 |  |  |  |  |  |
|  | *An. cinereus* |  | 1 |  |  |  |  |  |
| 13 | *An. arabiensis* | Larvae | 1295 | Dipping | Arjo-Dedessa irrigation site (Southwest Ethiopia) | Oromia | outdoor |  |
|  | *An. coustani,* |  | 152 |  |  |  |  |  |
|  | *An. pharoensis* |  | 76 |  |  |  |  |  |
|  | *An. squamosus* |  | 30 |  |  |  |  |  |
| 14 | *An. arabiensis* | Adult | 2150 | Light trap, PSC and pit trap | Ghibe River Basin (Guraghe Zone) | SNNP | Indoor/ outdoor |  |
|  | *An. coustani* |  | 130 |  |  |  |  |  |
|  | *An. pretoriensis* |  | 46 |  |  |  |  |  |
|  | *An. demeilloni* |  | 26 |  |  |  |  |  |
|  | *An. rupicolus* |  | 27 |  |  |  |  |  |
|  | *An. christyi* |  | 19 |  |  |  |  |  |
|  | *An. pharoensis* |  | 18 |  |  |  |  |  |
|  | *An. tenebrosus* |  | 17 |  |  |  |  |  |
|  | *An. ardensis* |  | 6 |  |  |  |  |  |
|  | *An. natalensis* |  | 1 |  |  |  |  |  |
|  | *An. ziemanni* |  | 1 |  |  |  |  |  |
|  | *An. rivulorum* |  | 2 |  |  |  |  |  |
| 15 | *An. arabiensis* | Larvae and adult | 871 | Dipping, light trap, PSC, pit trap and pot | Addis Zemen | Amhara | Indoor/outdoor |  |
|  | *An. cinereus* |  | 1561 |  |  |  |  |  |
|  | *An. demeilloni* |  | 75 |  |  |  |  |  |
|  | *An. christi* |  | 6 |  |  |  |  |  |
|  | *An. pretoreinsis* |  | 1 |  |  |  |  |  |
| 16 | *An. arabiensis* | Adult | 1153 | Human landing catches and light trap | Adami Tullu district (central Ethiopia) | Oromia | Indoor/outdoor |  |
|  | *An. pharoensis* |  | 342 |  |  |  |  |  |
|  | *An. ziemanni* |  | 5228 |  |  |  |  |  |
|  | *An. funestus* |  | 883 |  |  |  |  |  |
| 17 | *An. arabiensis* | Adult | 1914 | Light trap and PSC | Lare district, Nuer zone (south west Ethiopia) | Gambella | Indoor and outdoor |  |
|  | *An. pharoensis* |  | 602 |  |  |  |  |  |
|  | *An. nili* |  | 137 |  |  |  |  |  |
|  | *An. coustani* |  | 82 |  |  |  |  |  |
| 18 | *An. arabiensis,* | Larvae and adult | 487 | Dipping, light trap, aspirator and PSC | Zeway area (central Ethiopia) | Oromia | Indoor/outdoor |  |
|  | *An. pharoensis* |  | 467 |  |  |  |  |  |
|  | *An. coustani* |  | 365 |  |  |  |  |  |
|  | *An. cinereus* |  | 6 |  |  |  |  |  |
| 19 | *An. arabiensis* | Adult | 792 | Light trap | Seka-Chekorsa district, Jimma zone (south-west Ethiopia) | Oromia | Indoor/outdoor |  |
|  | *An. coustani* |  | 258 |  |  |  |  |  |
|  | *An. pharoensis* |  | 86 |  |  |  |  |  |
| 20 | *An. arabiensis* | Adult | 1119 | Light trap and PSC | Kersa district, Jimma zone (south-west Ethiopia) | Oromia | Indoor/outdoor |  |
|  | *An. pharoensis* |  | 98 |  |  |  |  |  |
|  | *An. coustani* |  | 342 |  |  |  |  |  |
| 21 | *An. arabiensis,* | Larvae and adult | 7663 | Dipping, light trap, aspirator and PSC | Metehara and  surrounding areas (eastern Ethiopia) | Oromia | Indoor/outdoor |  |
|  | *An. pharoensis* |  | 565 |  |  |  |  |  |
|  | *An. coustani .* |  | 30 |  |  |  |  |  |
|  | *An. ziemanni* |  | 5 |  |  |  |  |  |
|  | *An. ardensis* |  | 2 |  |  |  |  |  |
|  | *An. natalensis* |  | 1 |  |  |  |  |  |
| 22 | *An. arabiensis,* | Larvae | 450 | Dipping | Gamo (Arbamiinch)  Gofa Zone in the Great Rift Valley | SNPP | outdoor |  |
| 23 | *An. arabiensis,* | Larvae | 886 | Dipping | Between Adami Tulu and Meki towns (central Ethiopia) | Oromia | outdoor |  |
|  | *An. pharoensis* |  | 1015 |  |  |  |  |  |
|  | *An. squamosus* |  | 365 |  |  |  |  |  |
|  | *An. coustani* |  | 6 |  |  |  |  |  |
|  | *An. cinereus* |  | 7 |  |  |  |  |  |
| 24 | *An. arabiensis* | Larvae and adult | 62 | Dipping, Human biting catches, light trap and PSC | Akaki (Beseka) (Addis Ababa) | Oromia | Indoor |  |
|  | *An. christyi* |  | 1109 |  |  |  |  |  |
|  | *An. cinereus* |  | 245 |  |  |  |  |  |
|  | *An. pharoensis* |  | 5 |  |  |  |  |  |
|  | *An. coustani* |  | 1 |  |  |  |  |  |
| 25 | *An. cinereus* | Larvae and adult | 1176 | Dipping and light trap | Gondar Dembia ditrict, | Amhara | Indoor |  |
|  | *An. pharoensis* |  | 2 |  |  |  |  |  |
|  | *An. wilsonii* |  | 1 |  |  |  |  |  |
|  | *An. funestus* |  | 32 |  |  |  |  |  |
|  | *An. demeilloni* |  | 6 |  |  |  |  |  |
|  | *An. coustani* |  | 4 |  |  |  |  |  |
|  | *An. ziemanni* |  | 4 |  |  |  |  |  |
|  | *An. pretoriensis* |  | 6 |  |  |  |  |  |
|  | *An. arabiensis* |  | 1176 |  |  |  |  |  |
| 26 | *An. arabiensis* | Larvae and adult | 8104 | Dipping and light trap | Ziway area (central Ethiopia) | Oromia | Indoor and out door |  |
|  | *An. pharoensis* |  | 4494 |  |  |  |  |  |
|  | *An. coustani* |  | 1670 |  |  |  |  |  |
|  | *An. funestus* |  | 395 |  |  |  |  |  |
| 27 | *An. arabiensis* | Adult | 6903 | Light trap, PSC and pit trap | Sibu Sire district, East Wollega Zone (west Ethiopia) | Oromia | Indoor and out door |  |
| 28 | *An. zeimanni* | Adult | 2267 | human landing catches | Adami Tullu Jiddo Kombolcha district (south- central Ethiopia) | Oromia | Indoor and out door |  |
|  | *An. arabiensis* |  | 845 |  |  |  |  |  |
|  | *An. pharoensis* |  | 233 |  |  |  |  |  |
|  | *An. funestus* |  | 63 |  |  |  |  |  |
| 29 | *An. arabiensis* | Larvae and adult | 2246 | Dipping and light trap | Chafe, Ayetu and Toli in Sekoru district (south western Ethiopia) | Oromia | Indoor and out door |  |
|  | *An. squamosus* |  | 350 |  |  |  |  |  |
|  | *An. demeilloni* |  | 1139 |  |  |  |  |  |
|  | *An. garnhami* |  | 234 |  |  |  |  |  |
|  | *An. funestus* |  | 15 |  |  |  |  |  |
|  | *An. marshallii* |  | 67 |  |  |  |  |  |
|  | *An. longipalpis* |  | 54 |  |  |  |  |  |
|  | *An. pretoriensis* |  | 44 |  |  |  |  |  |
|  | *An. christyi* |  | 62 |  |  |  |  |  |
| 30 | *An. arabiensis* | Larvae | 602 | Dipping | Koka dam, Awash basin (south east Ethiopia) | Oromia |  |  |
|  | *An. pharoensis* |  | 1097 |  |  |  |  |  |
|  | *An. coustani* |  | 58 |  |  |  |  |  |
|  | *An. squamosus* |  | 38 |  |  |  |  |  |
| 31 | *An. arabiensis* | Adult | 1640 | HLC, PSC and CDC | Abaya, Bambasi (Asosa zone) and Lare (Gambela) | BenshanGumz and Gambella | Indoor/outdoor |  |
|  | *An. pharoensis* |  | 1000 |  |  |  |  |  |
|  | *An. cydippis* |  | 491 |  |  |  |  |  |
|  | *An. funestus* |  | 429 |  |  |  |  |  |
|  | *An. coustani* |  | 1949 |  |  |  |  |  |
|  | *An. ziemanni* |  | 375 |  |  |  |  |  |
| 32 | *An. gambiae* | Adult | 4660 | PSC, CDC and HLC | Arbaminch, Babile, Goro and Alamata | SNNP, Oromia and Tigray | Indoor/outdoor |  |
|  | *An. pharoensis* |  | 90 |  |  |  |  |  |
|  | *An. demeilloni* |  | 41 |  |  |  |  |  |
|  | *An. coustani* |  | 109 |  |  |  |  |  |
|  | *An. pretoriensis* |  | 1 |  |  |  |  |  |
|  | *An. maculipalpis* |  | 1 |  |  |  |  |  |
| 33 | *An. arabiensis* | Adult | 4249 | CDC | Chano (Arba Minch) | SNNP | Indoor |  |
|  | *An. marshalli* |  | 246 |  |  |  |  |  |
|  | *An. pharoensis* |  | 178 |  |  |  |  |  |
|  | *An. demeilloni* |  | 19 |  |  |  |  |  |
|  | *An. dancalicus* |  | 16 |  |  |  |  |  |
|  | *An. cinctus* |  | 14 |  |  |  |  |  |
|  | *An. culicifacies* |  | 12 |  |  |  |  |  |
|  | *An. funestus* |  | 10 |  |  |  |  |  |
|  | *An. obscures* |  | 7 |  |  |  |  |  |
|  | *An. tenebrosus* |  | 7 |  |  |  |  |  |
|  | *An. parensis* |  | 6 |  |  |  |  |  |
|  | *An. rufipes* |  | 5 |  |  |  |  |  |
|  | *An. ziemanni* |  | 4 |  |  |  |  |  |
|  | *An. garnhami* |  | 3 |  |  |  |  |  |
|  | *An. salbaii* |  | 2 |  |  |  |  |  |
